# Female first and senior authorship in high-impact critical care journals 2005–2024

**DOI:** 10.1186/s13054-025-05649-4

**Published:** 2025-09-08

**Authors:** Nora Bruns, Pia Brensing, Sandra Greve, Sandra Horsch, Ursula Felderhoff-Müser, Christian Dohna-Schwake, Simone Schwarz

**Affiliations:** 1 Department of Pediatrics I, University Hospital Essen, University of Duisburg-Essen, Hufelandstr, 55, Essen, 45239 Germany; 2 C-TNBS, Centre for Translational Neuro- and Behavioural Sciences, University Hospital Essen, University of Duisburg-Essen, Hufelandstr. 55, Essen, 45239 Germany; 3 Department of Neonatology, Helios Klinikum Berlin Buch, Schwanebecker Chaussee 50, Berlin, 13125 Germany

## Abstract

**Background:**

Gender disparities persist in medical research. This study assessed gender representation trends in first and senior authorships in the five highest-ranked critical care journals (by impact factor) over a 20-year period.

**Methods:**

We analyzed author gender distribution from 2005 to 2024. Author gender was determined using NamSor for web-based gender prediction. We assessed trends in female first, senior, and combined first and senior authorships by calculating percentages, and annual changes by linear regression for multiple and single author publications.

**Results:**

Among 42,970 articles, 34,743 had multiple authors and 8,227 had a single author. Despite progress over the past two decades, women remain underrepresented in critical care research leadership with 7.8% of publications having both female first and senior authors, compared to 56.7% with both positions held by men. Single authors were female in 23.6%. Linear regression showed increasing female authorships between − 0.1 and + 0.6% points per year depending on the journal, author position, and time period. Sensitivity analyses including only publications with more than 80% probability of correct gender classification yielded congruent results.

**Conclusions:**

Despite small but constant growth rates of female representation as first or senior authors in high impact critical care journals over the past 20 years, women remain clearly underrepresented. Given the current rate of change, it will take decades to achieve gender parity. The observed gender disparity in authorships likely reflects underlying gender inequities in critical care career trajectories, highlighting the need for institutional changes.

**Supplementary Information:**

The online version contains supplementary material available at 10.1186/s13054-025-05649-4.

## Background

Gender disparities in medical academia persist despite decades of awareness [1], potentially limiting the diversity of perspectives needed to advance patient care and scientific innovation. They are particularly evident in authorship positions associated with seniority and in prestigious journals [2]. Even more so, female first authorship in JAMA peaked at 38% of articles in 2011 and in NEJM at 28% in 2002 with current rates stagnating [3]. Besides being underrepresented as first authors, women take twice as long as men to transition to senior authorship [4] Notably, wealthy countries like Japan, Germany, and Switzerland have fewer female authors compared to lower resource settings [2].

This persistence of gender disparities occurs against a backdrop of global recognition that diverse research teams produce higher-quality and more innovative science. Recent analyses demonstrate that countries with progressive gender equity policies in research funding and editorial standards generate more reliable and clinically applicable findings [5]. The Sex and Gender Equity in Research (SAGER) guidelines emphasize that failure to conduct sex and gender-based analysis limits generalizability and applicability to clinical practice [6]. In critical care, where patient populations are highly diverse and complex, the underrepresentation of women in research leadership may limit the diversity of research questions, methodological approaches, and clinical perspectives needed to optimize outcomes across different groups of patients.

In critical care research, previously reported proportions of female first and senior authorship range around 30% for first authorship and between 16 and 20% for senior authorship [7, 8]. While the COVID-19 pandemic led to a surge in the number of critical care publications, the publication gender gap widened further [9]. The gender gap is even more pronounced in authorship for randomized controlled trials (RCT) in critical care [10] and for high-impact journals [8]. Publications from investigator-initiated research consortia show more equal gender distribution compared to general critical care medicine research [11, 12].

There is evidence on gender disparities in authorship in critical care literature, but studies covering two decades including pandemic and post pandemic years and focusing on all articles in the top-ranked journals listed in the critical care section of the journal citation reports are lacking. The aim of this meta-epidemiological study was to assess the most recent author gender representation and the dynamics of change over a twenty-year time period for first, senior and combined first and senior authorships in these journals.

## Methods

### Journal selection and data collection

We identified the top five critical care medicine journals based on 2023 the impact factor (IF) of the Journal Citation Reports via Web of Science (Clarivate Analytics, London, United Kingdom; accessed on November 2nd, 2024). PubMed entries from these journals from a 20-year period (January of 2005 - December of 2024) and full names of the first and senior authors were downloaded using Python software and the PubMed Application Programming Interface (API), which provides access to article metadata in the National Library of Medicine’s PubMed database (all data was retrieved on February 16th, 2025).

### Gender determination

We used NamSor (www.namsor.app, accessed November 3rd, 2024) to estimate the likely gender of each paper’s first and senior (= last) author based on their first and last names along with correct classification probabilities for each analyzed name. NamSor has been proven to have excellent overall performance [13] and comparably better performance on non-Western names than other gender-prediction algorithms [14].

### Data cleaning and handling of missing data

The results of the name analysis were merged with the article metadata and the dataset cleaned, which included:

- Identifying and removing articles without listed authors, e.g., abstract collections, retractions, and corrections.
- Detecting and removing articles where metadata extraction via the Pubmed API had technically failed, e.g. only first name initials were extracted instead of full first names.
- Drawing a 1% sample for human validation of the gender prediction algorithm from the cleaned main dataset.
- Creating a sensitivity analysis dataset that included only entries with a correct gender classification probability of ≥ 80%, because the NamSor algorithm provides a prediction for each name regardless of the certainty but provides information on the correct classification probability.

### Validation of the gender prediction algorithm

After drawing a random 1% sample from the final dataset, the 1% sample was split in half and each half was rated by two independent raters (half 1: PB, SG; half 2: SS, CDS) who were unaware of the gender prediction results with rating options “female”, “male”, and “undecided”. The raters used all available information, including common sense/experience and internet search of the specific authors.

### Outcomes measures

The main outcome was the percentage of each author combination per year for single and multiple author publications. The author combinations of interest were:

- First author female, senior author male.
- First author male, senior author female.
- First and senior author female.
- First and senior author male.

The secondary outcome was the annual change rate in percentage points per predefined time period (2005–2010, 2010–20015, 2015–2020, and 2020–2024; including start and end year) for each author combination.

### Statistical analyses

#### Descriptive analyses

Discrete variables are presented as counts and percentages, while continuous variables are presented as means for normally distributed data and medians for skewed distributions. Regression results are presented as the effect estimate and 95% confidence intervals (CI).

#### Validation

To validate the gender prediction results, Cohen’s kappa [15] and percent agreement were calculated for the pooled human ratings versus the algorithm. In addition, human versus human ratings were analyzed likewise to detect changes between raters. Ratings which had been rated as “undecided” by at least one rater were excluded from the analyses.

#### Main outcome

We calculated the annual frequencies and percentages of female and male first and senior authors separately and for the combination of both authors were female or male, for the overall dataset and separately for each journal. For single author publications, we calculated the annual frequencies and percentages for female and male authors. All analyses were additionally conducted for each journal.

#### Secondary outcome

For each of the author combination, annual change rates during the above-defined time periods were calculated using univariate linear regression for each respective period.

#### Sensitivity analysis

To minimize bias from potential inaccuracies in gender prediction, calculation of the main and secondary outcomes was repeated on a reduced dataset that included only publications with a probability of ≥ 80% correct classification for both the first and senior author or the single author, respectively.

#### Visualization of results

Besides representation of the results in tables and static figures, we created an interactive dashboard. The dashboard visualizes the frequencies or percentages for multiple and single author publications either overall or for the distinct journals based on user selections.

#### Missing data

Handling of missing metadata is described above. There were no missing data on author gender as determined by the algorithm. Because Lancet Respiratory Medicine was only established in 2013, analyses for this journal comprised only the period from 2013 to 2024.

### Software and statistical tools

We used Python 3.13 (Python Software Foundation, Beaverton, USA) in a Jupyter Lab environment (Version 4.3.4; Project Jupyter) [16] for API extraction of the publications and their metadata, data preparation, and data cleaning. Gender extraction was performed online with NamSor (https://namsor.app, NamSor SAS, Versaille, France) on February 16th, 2025. Statistical analyses and figure generation were performed using SAS Enterprise Guide on Demand for Academics Version 8.3 (SAS Institute, Cary, USA). The interactive dashboard was created as an.html document using JavaScript including additional libraries (jQuery, Plotly.js, and DataTables).

### Ethics approval

The study did not involve human subjects and therefore did not require ethical approval according to local legislation.

## Results

**Fig. 1:**
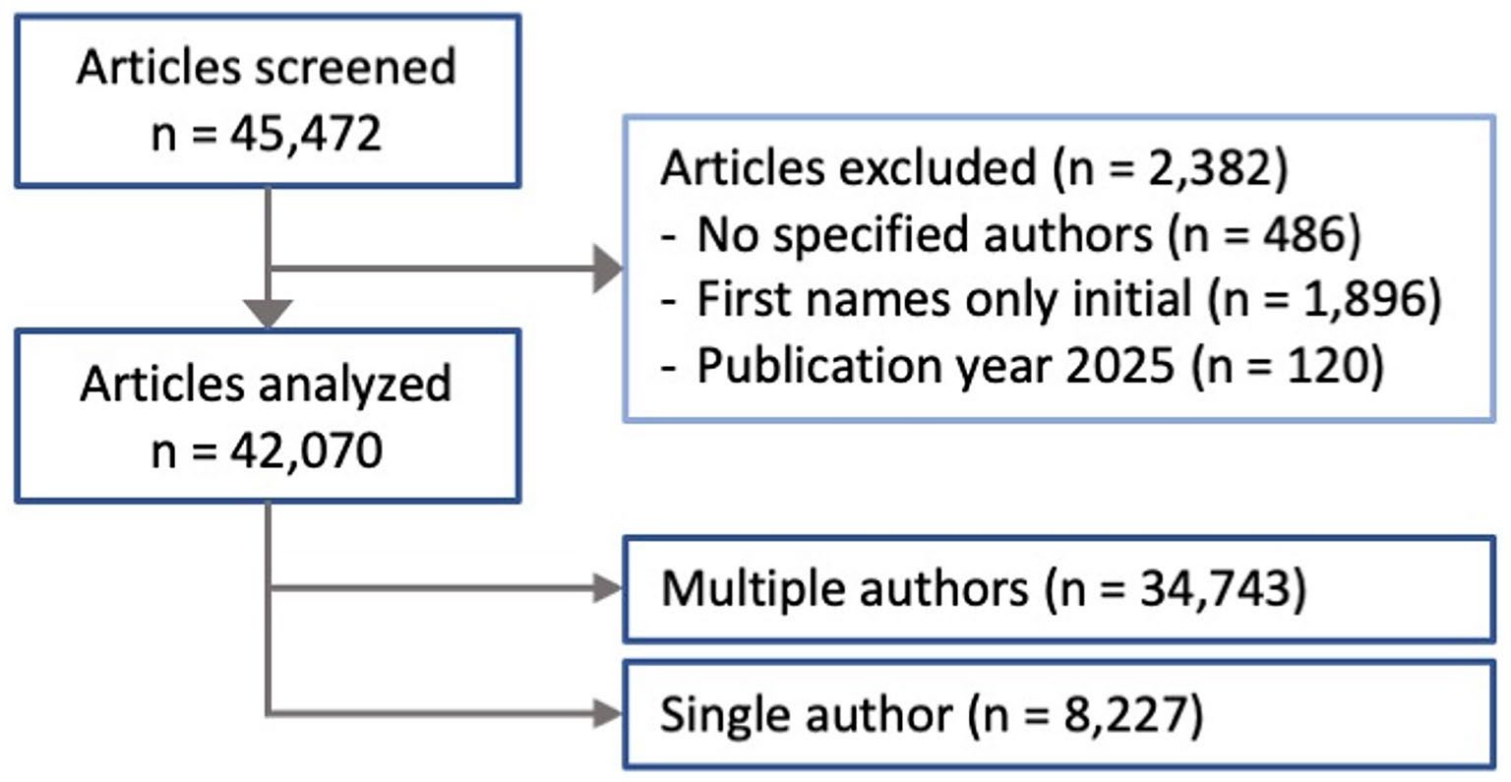
Article selection

The five highest-ranked journals in the 2023 Clarivate Journal Citation Reports for critical care medicine were *Lancet Respiratory Medicine* (IF: 38.7), *Intensive Care Medicine* (IF: 29.5), *American Journal of Respiratory and Critical Care Medicine* (IF: 19.3), *Chest* (IF: 9.5), and *Critical Care* (IF: 8.8). Out of 45,472 total entries published in these journals between January 2005 and December 2024, 486 articles without specified author were excluded, along with 1,896 entries where first names were only initials, and 120 entries with the publication date after December 31 st, 2024 (Fig. 1). Out of the remaining 42,970 files, 34,743 had multiple authors and 8,227 had a single author.

For the validation of the gender prediction algorithm, 430 records with 837 author entries from multiple and single author publications were analyzed, resulting in a total of 1674 human ratings. After exclusion of “undecided” ratings, 1504 valid ratings remained. The pooled human versus algorithm agreement was 94.3% (Cohen’s kappa 0.84) for first and senior authors. The pooled agreement was 93.6% for first authors (kappa 0.84) and 95.1% for senior authors (kappa 0.84). The inter-rater agreement was 96.8% (kappa 0.90) for rater 1 versus 2 and 96.4% (kappa 0.90) for rater 3 versus 4.

The median probability of correct gender classification across the first authors was 98% (10th to 90th percentile 78–99%) and 98% (84–99%) for senior authors. For single authors, the probability of correct classification was 98% (85–99%). There were no striking differences with respect to the probability of correct classification between the journals (Table 1).

**Table 1:** General information main analysis

	Overall	Lancet respiratory medicine*	Intensive care medicine	American journal of respiratory and critical care medicine	Chest	Critical care
**Multiple authors**						
Analyzed articles	34,743	1711	5,880	9,406	10,509	7,237
Probability of correct classification first author [%], *median (10th – 90th percentile)*	98 (78–99)	98 (84–99)	98 (84–99)	98 (78–99)	98 (79–99)	98 (71–99)
Probability of correct classification senior author, *median (10th – 90th percentile)*	98 (84–99)	98 (84–99)	98 (85–99)	98 (85–99)	98 (84–99)	98 (72–99)
First author female + senior author male, *n (%)*	8,012 (23.1)	381 (22.3)	1,174 (20.0)	2,367 (25.2)	2,534 (24.1)	1,556 (21.5)
First author male + senior author female, *n (%)*	4,353 (12.5)	263 (15.4)	621 (10.6)	1,272 (13.5)	1,400 (13.3)	797 (11.0)
Both authors female, *n (%)*	2,696 (7.8)	147 (8.6)	329 (5.6)	876 (9.3)	854 (8.1)	490 (6.8)
Both authors male, *n (%)*	19,682 (56.7)	920 (53.8)	3,756 (63.9)	4,891 (52.0)	5,721 (54.4)	4,394 (60.7)
**Single authors**						
Analyzed articles	8,227	1,553	1,056	2,083	2,185	1,350
Probability of correct classification [%], *median (10th – 90th percentile)*	98 (85–99)	98 (65–99)	98 (87–99)	98 (89–99)	98 (86–99)	98 (84–99)
Female, *n (%)*	1,945 (23.6)	566 (36.4)	191 (18.1)	451 (21.7)	444 (20.3)	293 (21.7)

*Since its foundation in 2013

In multiple-author publications, 8,012 (23.1%) had a female first and male senior author, whereas 4,353 (12.5%) had the distribution vice versa. Female first and senior authors were identified in 2,696 (7.8%) publications, whereas male first and senior authors comprised 19,682 (56.7) publications. Among single authors, 1,945 (23.6%) were identified as female. These findings were largely similar between the journals with higher variation among single authorship compared to multiple author publications (Table 1).

**Fig. 2:**
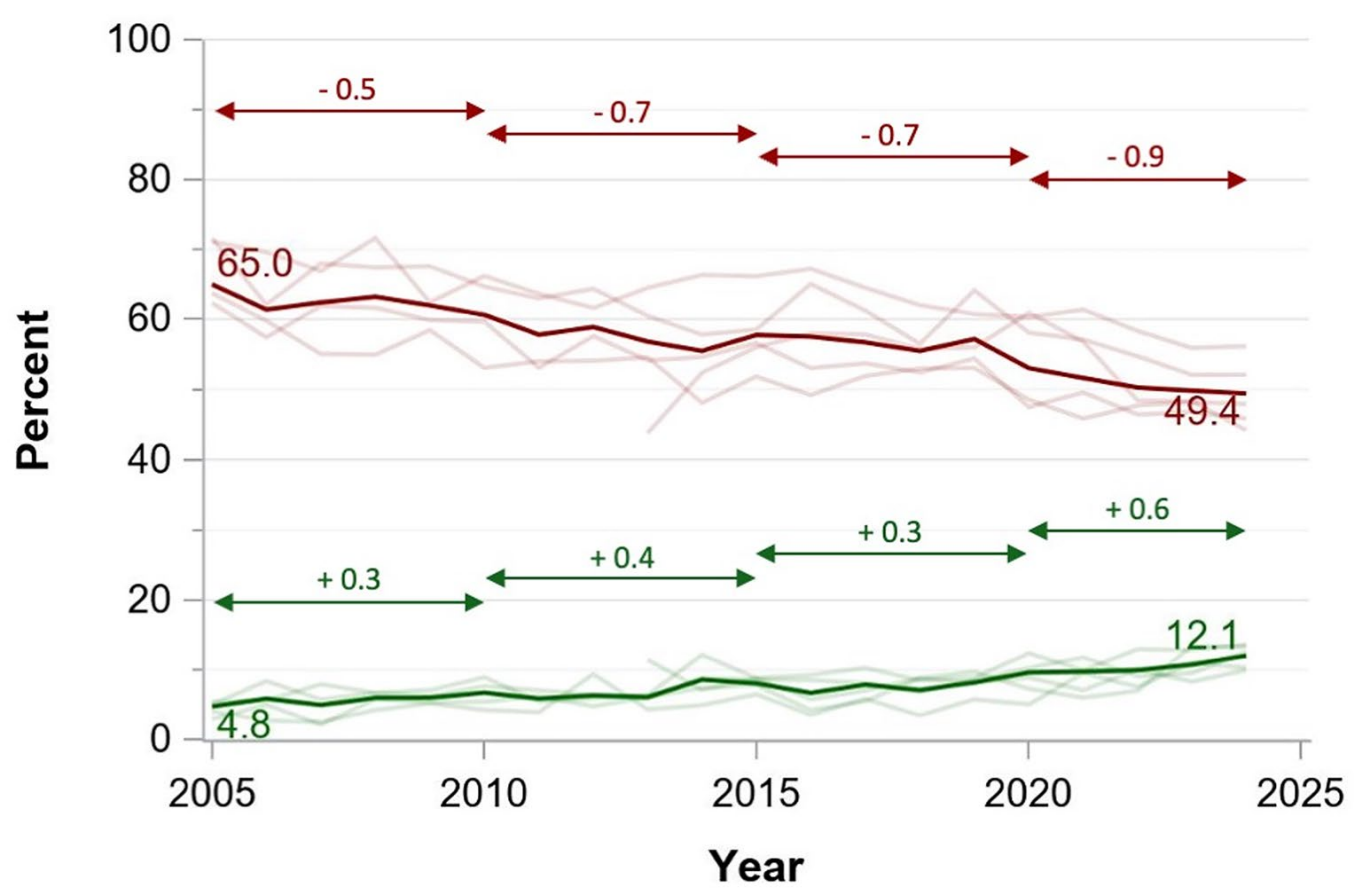
Evolution of authorship combinations between 2005 and 2024 in the five highest-impact critical care journals (%) for first and senior authors with distinct gender *Blue solid line*: Publications with first author female and senior author male. *Red solid line*: Publications with first author male and senior author female. *Pale blue and green lines*: Percentages by journal. *Double-headed* arrows indicate periods for linear regression to calculate annual change rates (*numbers above the arrow lines*)

**Fig. 3:**
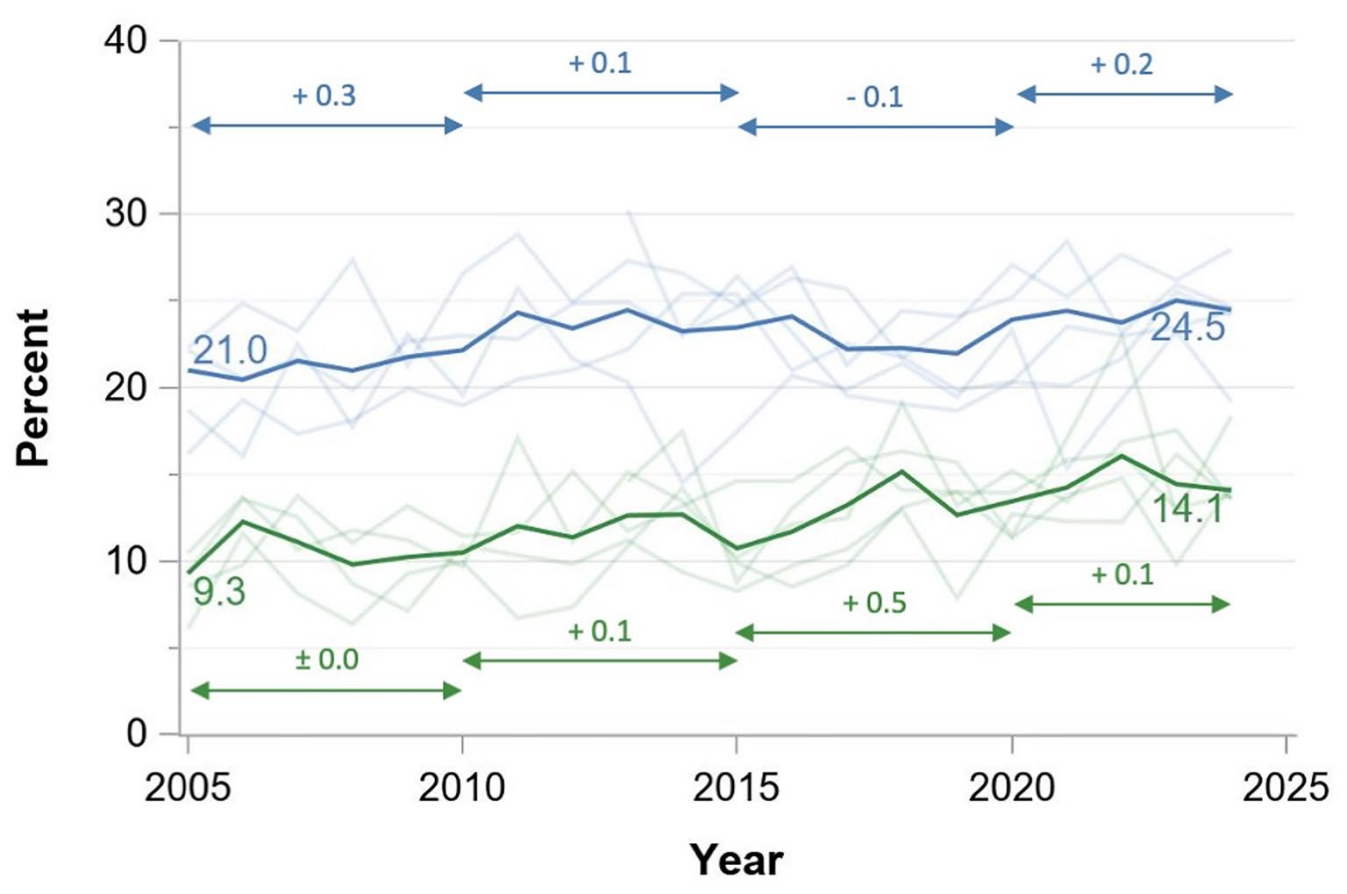
Evolution of authorship combinations between 2005 and 2024 in the five highest-impact critical care journals (%) for first and senior authors of the same gender *Green solid line*: Publications with first and senior author female. *Red solid line*: Publications with first and senior author male. *Pale red and green lines*: Percentages by journal. *Double-headed* arrows indicate periods for linear regression to calculate annual change rates (*numbers above the arrow lines*)

Publications with female first and male senior authors increased from 21.0 to 24.5% from 2005 to 2024 and publications with male first and female senior authors increased from 9.3 to 14.1%. While publications with both female authors increased from 4.8 to 12.1% between 2005 and 2024, men still dominated double-authorship positions with 49.4% of all publications in 2024. Female single authorship increased from 11.7 to 30.8% with male authorship declining symmetrically (Figs. 1, 3 and 2 and interactive dashboard in the supplementary online material).

**Fig. 4:**
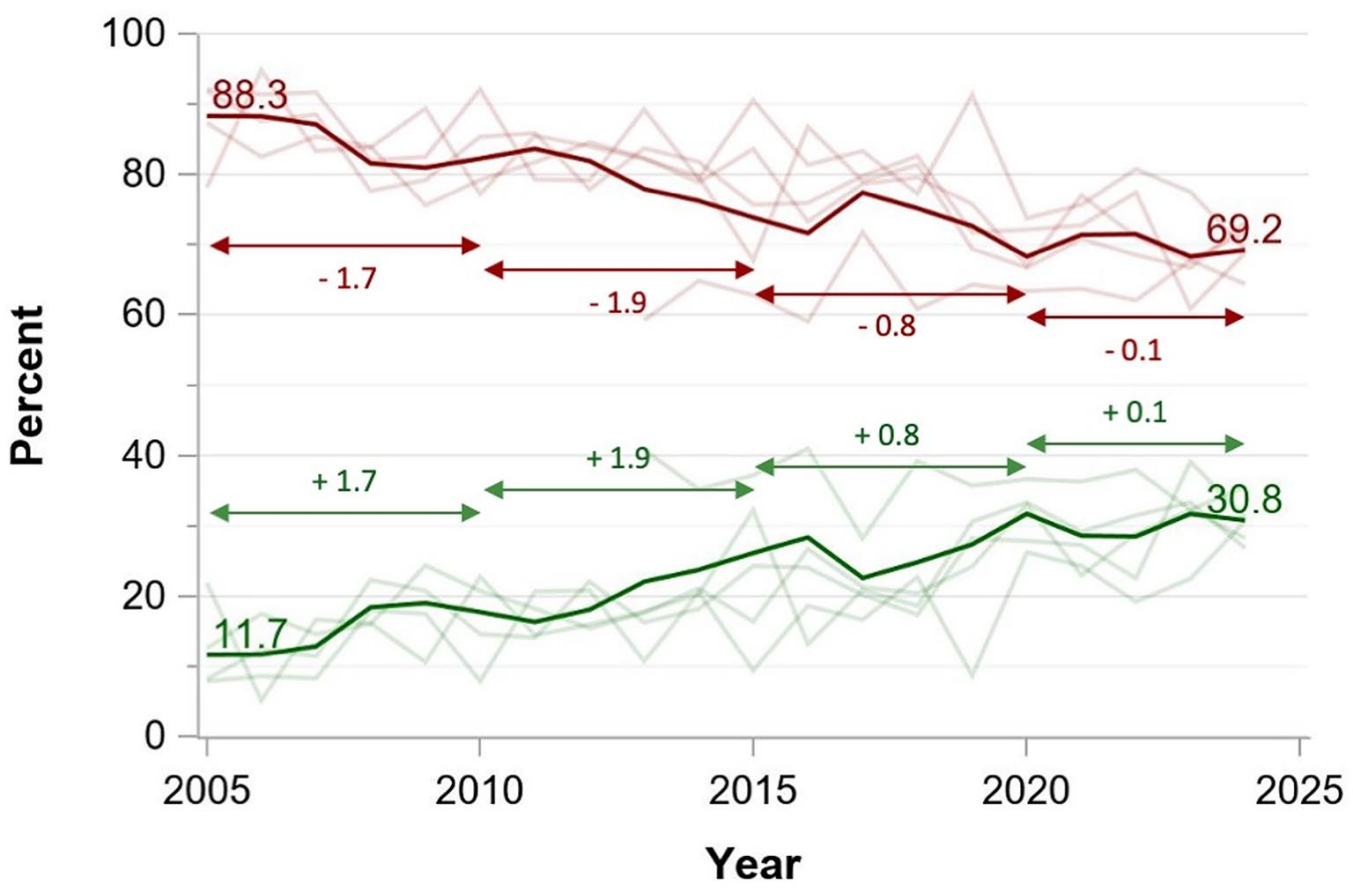
Evolution of single authorship gender distribution between 2005 and 2024 in the five highest-impact critical care journals (%) *Green solid line*: Author female. *Red solid line*: Author male. *Pale red and green lines*: Percentages by journal. *Double-headed* arrows indicate periods for linear regression to calculate annual change rates (*numbers above the arrow lines*)

Linear regression showed increasing female authorships over time with recent increase in annual change rates for publications with female first and senior authors (Fig. 3), publications with male first and female senior authors (Fig. 2), publications with single authors (Fig. 4). The annual increase rates varied between − 0.1 and 0.6% points per year depending on the author combination. These proportions were consistent across the study period, with all journals showing similar patterns of gender distribution in authorship positions.

For the sensitivity analysis, 5,368 records were discarded due to a probability of correct gender classification below 80% (first author: *n* = 2,445; senior author: *n* = 1,706; both authors: *n* = 1,217; single author: *n* = 626). The remaining 36,976 publications (29,375 multiple authors; and 7,601 single authors) were analyzed analogously to the main analyses. The probability of correct classification was equal in the initial cohort with respect to median and 90th percentiles and higher for the 10th percentiles (Table 2). Lower proportions of female authors for multiple and single author publications were observed for all types of female author constellations overall and across journals, with *Lancet Respiratory and Critical Care Medicine* as the only exception, where the proportion of female authorships was higher in the sensitivity analysis (Table 2). Annual change rates were similar as in the main analysis for multiple author combinations and for single author publications (Table 3).

**Table 2:** Sensitivity analysis - general information

	Overall	Lancet respiratory medicine	Intensive care medicine	American journal of respiratory and critical care medicine	Chest	Critical care
**Multiple authors**						
Analyzed articles (≥ 2 authors)	29,375	1,504	5,135	7,992	8,897	5,847
Probability of correct classification first author [%], *median (10th – 90th percentile)*	98 (92–99)	98 (92–99)	98 (93–99)	98 (92–99)	98 (91–99)	98 (93–99)
Probability of correct classification last author, *median (10th – 90th percentile)*	98 (93–99)	98 (91–99)	98 (94–99)	98 (94–99)	98 (93–99)	98 (94–99)
First author female + last author male, *n (%)*	6,497 (22.1)	328 (21.8)	979 (19.1)	1,999 (25.0)	2,051 (23.1)	1,140 (19.5)
First author male + senior author female, *n (%)*	3,324 (11.3)	220 (14.6)	502 (9.8)	995 (12.4)	1,069 (12.0)	538 (9.2)
Both authors female, *n (%)*	2,015 (6.9)	117 (7.8)	246 (4.8)	713 (8.9)	655 (7.4)	284 (4.9)
Both authors male, *n (%)*	17,539 (59.7)	839 (55.8)	3,408 (66.4)	4,285 (53.6)	5,122 (57.6)	3,885 (66.4)
**Single authors**						
Analyzed articles	7,601	1,346	986	1,987	2,043	1,239
Probability of correct classification [%], *median (10th – 90th percentile)*	98 (90–99)	98 (90–99)	98 (94–99)	98 (93–99)	98 (92–99)	98 (93–99)
Female, *n (%)*	1,593 (20.9)	399 (29.6)	159 (16.1)	411 (20.7)	388 (19.0)	236 (19.0)

**Table 3:** Sensitivity analysis – annual change rates in percentage points for authorship combinations

	Year			
	2005–2010	2010–2015	2015–2020	2020–2024
**Mutiple authors**				
First author female + last author male	0.4 (0.0–0.8)	0.1 (−0.4–0.7)	0.0 (−0.6–0.6)	0.0 (−0.5–0.5)
First author male + senior author female	−0.1 (−0.9–0.7)	0.1 (−0.5–0.8)	0.4 (−0.4–1.2)	0.0 (−1.0–1.0)
Both authors female	0.2 (0.0–0.4)	0.4 (0.1–0.7)	0.2 (−0.3–0.7)	0.8 (0.3–1.2)
Both authors male	−0.5 (−1.8–0.8)	−0.6 (−1.4–0.1)	−0.6 (−1.1–0.0)	−0.8 (−1.6–0.1)
**Single authors**				
Female	1.5 (0.1–2.9)	1.4 (0.7–2.2)	1.1 (−1.1–3.3)	0.0 (−2.6–2.5)
Male	−1.5 (−2.9 – −0.1)	−1.4 (−2.2 – −0.7)	−1.1 (−3.3–1.1)	0.0 (−2.5–2.6)

## Discussion

This study on author gender representation in the five highest-impact critical care journals over the past 20 years found noticeable improvements but yet a persisting underrepresentation of women in first and senior authors roles. While men held the first and senior author positions in more than half of the publications, female first and senior authors accounted for less than 10% of all publications. Approximately 35% of all publications had mixed gender first and senior authors. For single author publications, female authorship also improved and reached approximately 30% in 2024. The annual rate of increase for any type of female authorships was below 1% point, showing slightly higher annual increases in recent years compared to the beginning of the observation period.

In medicine overall, strong progress has been made since the 1970 s [1], but in spite of gender parity among medical school graduates, there seems to be stagnation in recent years at around 30% for female first authorships as well as for representation in leadership positions [3, 17, 18]. Regarding the field of critical care, similar proportions and growth rates as in our study have been reported previously on shorter time periods and on selected subsets of publications [7, 8, 10]. Despite a growing female critical care workforce, women remain underrepresented for leadership positions, in medical societies, editorial boards, guideline committees, and as speakers at conferences [19–26]. Thus, the here-presented study likely depicts a symptom with multifaceted underlying factors.

Even though gender disparities are not limited to critical care, acknowledged barriers such as implicit bias, work-life balance challenges, mentorship gaps, and limited access to networking and funding opportunities might be exacerbated by the demanding and high-stress nature of critical care medicine. As a result, critical care is among the fields with the lowest representation of female physicians in the work force with 20 to 40% [27]. After correcting for the proportion of the work force, women’s representation in leadership positions is still lower, translating into only 20% of female faculty in the field of critical care in Canada, and only 37% female members of critical care societies worldwide [27, 28]. Along with slower transition of female first authors to senior authors compared to men [4], these structural barriers create a self-reinforcing cycle that consolidates leadership gaps [21]. The implications of gender disparities in the field of critical care are far-reaching: Underrepresentation of women in research leadership may limit the diversity of research questions, methodological approaches, and clinical perspectives that are needed to optimize care for all patients [29, 30]. These priorities and recommendations translate into medical practice not tailored to the patient’s sex, as has been acknowledged for several fields of medical research in general [31, 32] and critical care in particular [33–35]. Besides promoting diversity and equity, increasing female representation in research leadership might also enhance scientific integrity: retraction rates of articles authored by women are slightly below their overall representation in biomedical research, especially for reasons related to fraud or misconduct [36].

Contrasting these persisting inequities, international research consortia such as the Pediatric Acute Lung Injury and Sepsis Investigators (PALISI) network and the Canadian Critical Care Trials Group have shown that actively pursuing gender equality in scientific publications can effectively increase female representation among authors [11, 12]. Further, in the subfield of pediatric intensive care publications the gender gap is less pronounced for first authorships than in adult intensive care but equal for last authorships [37]. For pediatric RCTs, female authorship still lags behind the proportional female work force but has increased over time [38].

**Fig. 5:**
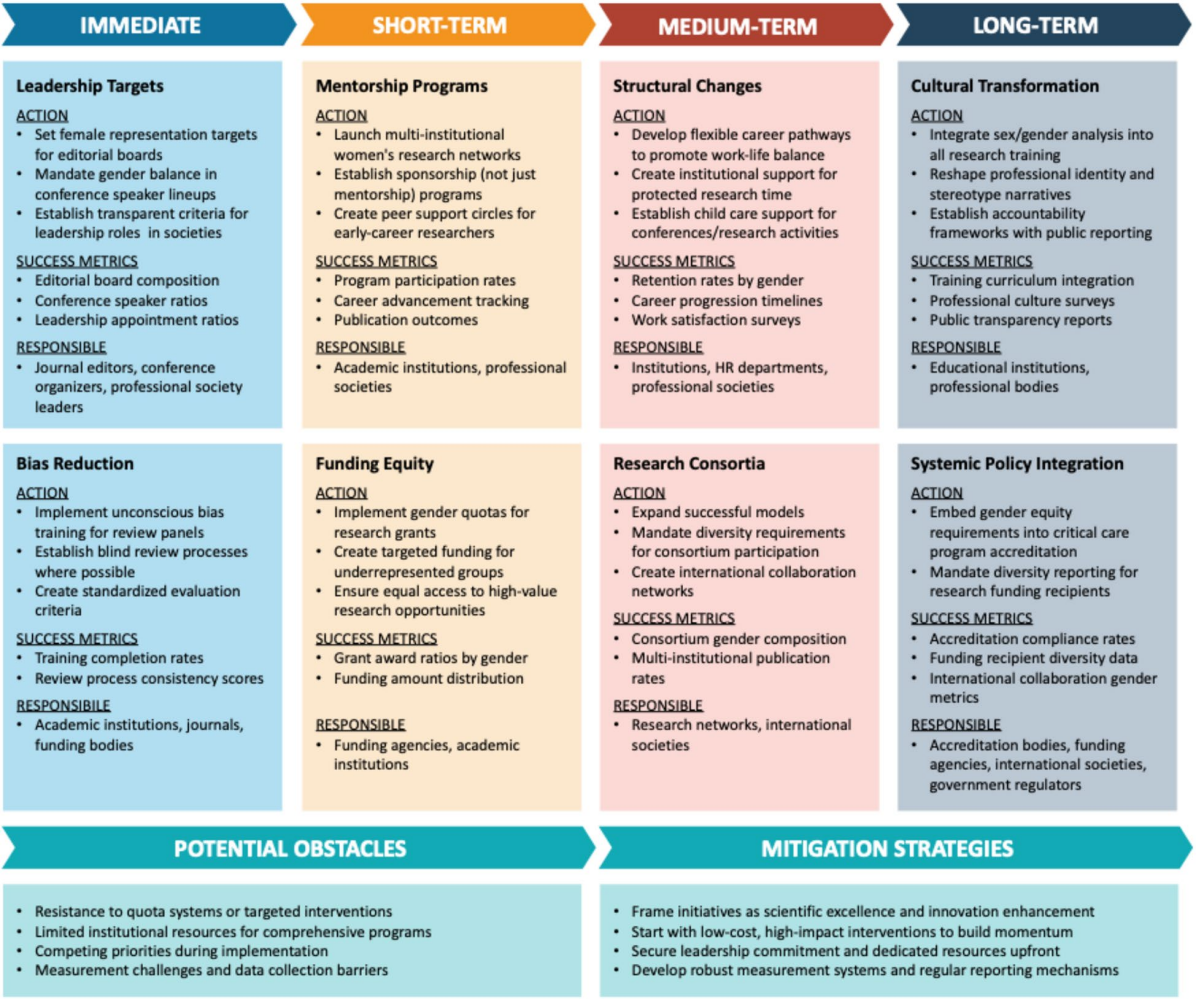
Phased implementation strategy for advancing gender equity in critical care medicine

These encouraging findings suggest that critical care societies and institutions could reduce gender disparities in research roles, leadership, and career development by implementing institutional changes as outlined, e.g., by Yong et al. [39]. Major fields of action outlined include increasing visibility of female role models, implementing targets for female representation, embracing sponsorship of women, employing transparent selection processes, undertaking unconscious bias training, and creating flexible working environments. Striving for inclusive leadership, providing institutional support, facilitating collaboration through advocacy groups, and engaging in men in the process may further enhance improvement [39]. Similar claims including action plans or blueprints have been published by various authors [23, 40–43], possibly reflecting the broadly perceived need for urgent change accompanied by the impossibility to effectively drive measurable change as an individual. A recent position paper by the “International Women in Intensive and Critical Care Medicine” network introduced a new aspect to the discussion: society’s need to challenge and reshape stereotypes about who can be a scientist [44]. While there is no doubt that policy changes and action plans at multiple levels are needed to address and remove organizational, institutional, structural and systemic barriers [45], some specific interventions and programs have yielded measurable success (Table 4) [46–51]. Notably, all of these successful programs included multi-faceted interventions aiming at more than one aspect, highlighting the complexity of the required changes. Given the slow pace of current progress documented in our analysis, Fig. 5 integrates these evidence-based strategies and successful interventions into a systematic implementation framework that prioritizes high-impact interventions and coordinates efforts across institutional levels to accelerate change.

**Table 4:** Successful interventions to promote gender equity

Type of intervention	Example
Institutional programs	**Women in Medicine and Health Science program at University of California**,** Davis** [46]: - Multilevel approach to promote networking, interaction, and collaboration among females - Female faculty increased from 18–36% within 10 years; female department chairs increased from 5–23%.
Mentorship	**Female Global Scholars Program (part of Women in Global Health Research at Weill Cornell Medicine)** [47]: - Multifaceted low-cost approach including two in-person symposia with workshops and regular webinars fort wo years - Six out of 10 participants received academic promotions by the end year one; the 10 scholars collectively presented at 11 conferences and submitted 22 abstracts and 19 manuscripts.
Funding quotas	**National Health and Medical Research Council (NHMRC) in Australia** [48]: - special measures under the Sex Discrimination Act, aiming to award equal numbers of grants to women and men - after two funding cycles, there was equal awarding of grants to women and men
Organizational initiatives	**Athena SWAN Charter (Scientific Women’s Academic Network) by the Equality Challenge Unit** [49, 51]: - provides institutional accreditation for gender equity efforts in higher education and research institutions - used for self-assessment and benchmarking regarding gender-sensitive policies, and measurable progress on representation and academic promotion - Athena SWAN members showed greater and faster growth in female representations, with higher award levels showing faster progress

Limitations of our study include the challenges in gender determination and the selection of journal articles. The automated assessment allowed processing of a large number of articles but may have caused misclassification, especially of Chinese or non-western names [14]. The risk of misclassification was addressed by human validation of a 1% sample which found almost perfect human-algorithm and inter-human agreement according to the recommended interpretation of Cohen’s kappa [15]. The results from the sensitivity analysis that excluded all articles with a probability of correct classification < 80% were congruent with the main analysis but may have introduced bias towards a higher proportion of western authors. Shared first or senior authorships could not be identified by our automated approach. By analyzing only the five highest impact journals due to capacity limits in this unfunded study, this analysis may have missed current trends in medium- or low-impact journals. Further, in large parts of Europe and some other countries in the world, there is institutional overlap between anesthesia and intensive care, potentially limiting the generalizability of the results for settings with strict separation of the two fields, as indicated by a systematic review that found lower parity in intensive care medicine compared to anesthesiology [52].

## Conclusion

Despite the constant small gains in female authorships, this study highlights that underrepresentation of female first and senior authors in today’s critical care literature persists and that the dynamics of change are small. Given that current change rates would require decades to achieve parity, critical care institutions must implement immediate, systematic multi-layered interventions to accelerate progress and harness the full spectrum of scientific talent needed to advance patient care.

## Supplementary Information

Supplementary Material 1.

## Data Availability

The dataset generated for the study will be made available to any qualified researcher upon request.
