# Supplementary material for: Female first and senior authorship in high-impact critical care journals 2005–2024
Source: Crit Care. 2025 Sep 8;29:395. doi: 10.1186/s13054-025-05649-4 (PMC12418694; doi:10.1186/s13054-025-05649-4)
Supplement: Supplementary file 1 — Supplementary Material 1. [file 13054_2025_5649_MOESM1_ESM.html]

Publication Dashboard


Publication Dashboard


- Dashboard
- Summary

About this Dashboard

This dashboard visualizes the gender distribution of first and last authors in scientific publications from 2005 to 2024.

##### Gender Combinations:

- **male-male**: Male first author, male last author
- **female-male**: Female first author, male last author
- **male-female**: Male first author, female last author
- **female-female**: Female first author, female last author

##### Journal Abbreviations:

- **AJRCCM**: American Journal of Respiratory and Critical Care Medicine
- **Chest**: Chest
- **Critical Care**: Critical Care
- **ICM**: Intensive Care Medicine
- **Lancet RM**: The Lancet Respiratory Medicine


### -

Total Publications (2024)

### -

% Female First Authors

### -

% Female Last Authors

### -

% Female First & Last Authors

Filters

Journal

Gender Combination

Reset Filters

Multiple author publications by gender combination and journal over time

Percentage
Total

Single author publications by gender and journal over time

Percentage
Total


Summary

#### Take-home message:

- In high-impact critical care journals, women represented only one-third of first authors and less than one fifth of senior authors between 2005 and 2024.
- The gender gap was most pronounced in publications by female author pairs (7.8 %) compared to male author pairs (56.7 %).
- At the current slow rate of progress, it will take decades to achieve gender parity in critical care research authorship without targeted interventions.


© 2025 Female Authorship in Critical Care Publications - Dashboard
